# Influence of Occupation on the Developement of Phthisis Pulmonatis

**Published:** 1831

**Authors:** 


7. *Influence of occupation on the development of Phthisis Pulmonalis.*—At a meeting of the French Institute, in November, 1830, M. M. Dumeril and Majendie made report on a work of M. Benoiston de Chateaneuf, relative to influence of professions in the development of pulmonary consumption. From an account of patients admitted into four hospitals, from 1817 to 1827, the following results were obtained:

1st. *Occupations which expose the lungs to the action of air charged with vegetable particles.* These are starchmakers, bakers, colliers, rag-dealers, cotton-spinners, winders, and silk-reelers.

Of 5829 males, 182 died.
Of 2555 females, 56 died.

Total, 8384. 158 died.

2d. *Occupations which expose the lungs to an air charged with mineral poisons.* These are quarriers, masons and their assistants, stonecutters, plasterers, &c.

Of 5829, 114 died.

3. *Occupations which expose the lungs to an air charged with animal particles.* These are brushmakers, carders, mattrassmakers, hatters, workers in feathers, &c.

Of 1434 males, 64 died.
Of 795 females, 27 died.

Total, 2229, 91 died.

4. *Occupations exposing the lungs to an air charged with offensive vapors.* These are gilders, painters, and the like.

Of 3337, 105 died.

5. *Occupations which expose the body, and especially the lower extremities to the action of moisture.* These are bleachers and washerwomen.

Of 2993, 129 died.

6. *Occupations which expose the muscles of the breast and the upper extremities to sudden and very severe exercise.* These are weavers, players, carpenters, cabinet-makers, smiths, farriers, water-carriers, sawers of wood and stone.

Of 554, 120 died.

7. *Occupations which subject the muscles of the breast and arms to constant motion, and the body to a bent position.* These are accountants, jewellers, tailors, shoemakers, fringemakers, lacemakers, lapidaries, workers in embroidery, florists, gloviers, &c.

Of 15,583, 837 died.

Total number of the phthisical patients of the various occupations above named, - - - - 43,001

Total number of deaths in ten years, - 1654.

*Journ. de Chim. Med. Jan.* 1831.

